# Safety and feasibility of total laparoscopic radical resection of Siewert type II gastroesophageal junction adenocarcinoma through the left diaphragm and left thoracic auxiliary hole

**DOI:** 10.1186/s12957-021-02183-9

**Published:** 2021-03-13

**Authors:** Yun Huang, Gang Liu, Xiumei Wang, Yan Zhang, Guijun Zou, Zhanwei Zhao, Zhen Cao, Huibin Zhao, Xinpu Yuan, Chaojun Zhang

**Affiliations:** General Surgery, Sixth Medical Center, PLA General Hospital, Beijing, 100048 China

**Keywords:** Adenocarcinoma of the esophagogastric junction, Siewert type II, Laparoscopic surgery, Minimally invasive treatment

## Abstract

**Background:**

The incidence of adenocarcinoma of the esophagogastric junction (AEG) is rising every year; however, the mode of operation for Siewert II AEG is still controversial. Accumulating evidence has shown that transabdominal surgery is better than transthoracic surgery for Siewert II AEG with esophageal invasion < 3 cm. In patients with obesity, a large tumor size, and high transection of the esophagus, the transabdominal esophageal hiatus approach for lower mediastinal lymph node dissection and posterior mediastinal anastomosis is difficult. Thus, total laparoscopic radical resection of Siewert II AEG is carried out through the left diaphragm and left chest auxiliary hole for the optimal surgical field of vision and space. In this prospective study, we assessed the feasibility of carrying out the procedure abdominally through the left diaphragm and auxiliary hole.

**Methods:**

Ten patients with Siewert II AEG were recruited between April and June 2019. Siewert II AEG was treated by total laparoscopy through the left diaphragm and left chest auxiliary hole. Clinicopathological features, surgical data, and adverse events were collected and analyzed in this prospective study.

**Results:**

The average duration of the operation was 348 ± 37.52 min, lower mediastinal dissection took 20.6 min, the OrVil anastomosis time was 29.8 min, the time necessary to suture the seromuscular layer through the left thoracic auxiliary hole was 11 min, the safety margin was 3.2 cm, and the total number of lymph nodes dissected was 40.6. The number of lower mediastinal lymph nodes dissected was 6.2. The rate of lymph node metastasis in the N110 group was 9 ± 12.45%, and the average intraoperative blood loss was 170 ± 57.47 mL. No anastomotic leakage or anastomotic stricture occurred after the operation. The time of intestinal function recovery was 2 days, and the first time of enteral nutrition through a jejunal nutrition tube was 2.4 days. No tumor recurrence was found in 10 patients at 1 year postoperatively.

**Conclusion:**

Total laparoscopic radical resection through the left diaphragm and left thoracic auxiliary hole for Siewert II AEG patients is feasible and safe. Thus, it may be a good surgical alternative for patients with esophageal tumors invading less than 3 cm.

**Trial registration:**

ChiCTR, ChiCTR2000034286. Registered 8 July 2020, http://www.chictr.org.cn/showproj.aspx?proj=55866.

**Supplementary Information:**

The online version contains supplementary material available at 10.1186/s12957-021-02183-9.

## Background

In recent years, the morbidity of esophagogastric junction (EGJ) cancer has increased every year worldwide, and the proportion of patients with adenocarcinoma of the esophagogastric junction (AEG) is significantly higher than before. According to statistics, approximately 80% of esophageal cancer cases in the world occur in underdeveloped areas, while 59% of new AEG patients reside in East/Southeast Asia [1]. Despite their rising incidence, there is still no consensus on the best surgical approach for these tumors due to narrow space, digestive tract reconstruction, anastomotic leakage, and pulmonary infection.

Modifications have been suggested to improve outcomes, with transabdominal techniques showing promising results. The JCOG9502 study by Kurokawa et al. [2] showed that the incidence of postoperative pneumonia in the transthoracic group was significantly higher than that in the transabdominal group (13% vs. 4%, *P*=0.048). The 5-year overall survival rates of patients in the transthoracic and transabdominal groups were 37.9% and 52.3%, respectively. The 10-year follow-up results were similar to those described above, with overall survival rates of 37% and 51% in the transthoracic and transabdominal groups, respectively (*P*=0.060). Therefore, for Siewert type II and type III patients with esophageal invasion < 3 cm, the transabdominal approach reduces the incidence of postoperative complications and improves the long-term prognosis [2].

In 2015, Takiguchi et al. reported 6 cases of laparoscopic incision of the left diaphragm for Siewert II AEG [3]. Since 2017, our center has carried out complete laparoscopic radical surgery for Siewert II AEG via the left diaphragm approach and accumulated experience. However, if the tumor location is high, reconstruction of the digestive tract through transabdominal transection of the esophagus and thorax is challenging. Therefore, based on the original surgical procedure, our center added a 12-mm auxiliary hole between the 6th and 7th left intercostals to overcome these issues. Thus, this technique is simple and effective for lower mediastinal lymph node dissection and digestive tract reconstruction and can greatly reduce the difficulty of the operation, shorten the duration of the operation, and improve the safety of the procedure. Herein, we introduce this method and provide short-term results.

## Materials and methods

### Indications

The subjects in this study had stages II and III Siewert II AEG. The clinical tumor depth was T2–T4, and no distant metastasis or upper and middle mediastinal lymph node enlargement was detected on the preoperative imaging examination.

All procedures carried out in this study were in accordance with the ethical standards of the institutional and national responsible committee on human experimentation and the Helsinki Declaration of 1964 and its later amendments or equivalents. This study was approved by the Ethics Committee of the Sixth Medical Center, PLA General Hospital. Informed consent was obtained from all individual patients included in the study. The clinical trial registration number is ChiCTR2000034286 (Chinese Clinical Trial Registry).

### Surgical steps

#### Posture and standing position (Fig. 1a)

The patient was placed in the supine position with his/her legs apart, and the head was placed at a height of approximately 45–60°. The operator was on the left side of the patient, the first assistant was on the right side of the patient, and the laparoscopic assistant was standing between the patient’s legs.

**Fig. 1:**
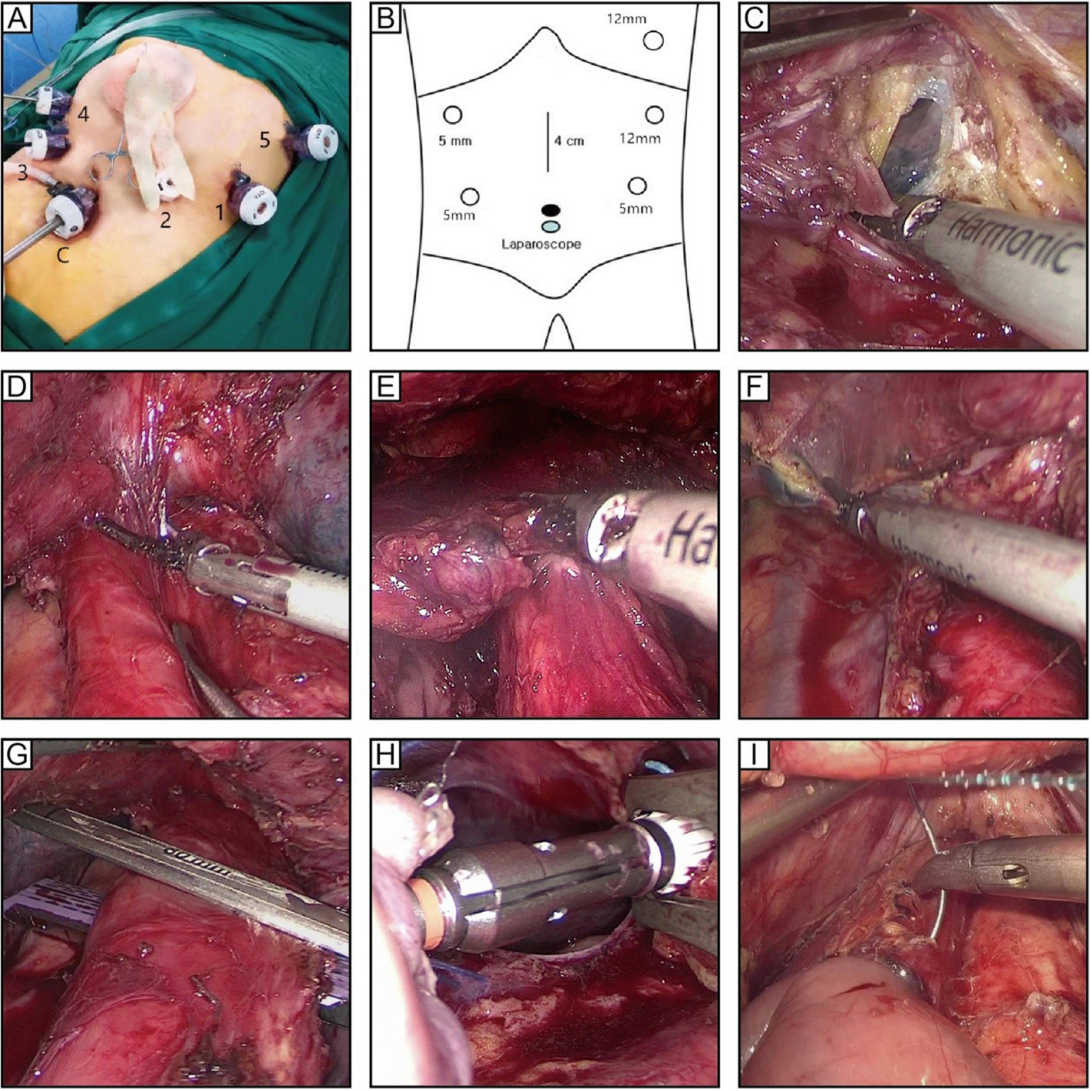
**a** Posture. **b** Trocar distribution. **c** The left diaphragm and left pleura were incised. **d** Bilateral inferior pulmonary veins were exposed by dissociation. **e** Lymph node dissection in lower mediastinum No. 110. **f** Lymph node dissection in lower mediastinum No. 112. **g** The esophagus was removed through the auxiliary foramen of the left chest. **h** Intrathoracic esophagojejunostomy with OrVil under direct vision. **i** Continuous suture of the seromuscular layer of anastomosis

#### Location of the trocar hole (Fig. 1b)

The lens hole was located under the umbilical cord, and hole 1 (12 mm) was located 2 cm below the costal edge of the left anterior axillary line. Hole 2 (5 mm) was located on the umbilical cord of the midline of the left clavicle (2 cm). Hole 3 (5 mm) was located on the umbilical cord of the midline of the right clavicle (2 cm). Hole 4 (5 mm) was located 2 cm below the costal margin of the right anterior axillary line. Hole 5 (12 mm) was located between the 6th and 7th intercostals of the right anterior axillary line.

An additional movie file shows this process in more detail (see Additional file 1).


**Additional file 1: Video.**

The superior mesenteric vein was exposed from the inferior margin of the pancreas, and the lymph nodes of the superior mesenteric vein (No. 14V) were explored. The right gastroepiploic artery and vein were removed. The subpyloric lymph nodes (No. 6) were dissected.

The dorsal membrane of the pancreas was dissociated to expose the gastroduodenal artery, which was separated from the distal end to the proximal end towards the proper hepatic artery. After an incision was made in the vascularized area between the proper hepatic artery and the duodenum (the gastric tube was withdrawn to 35 cm), the duodenum was removed with an intracavitary linear cutting occluder.

From right to left, the right gastric artery was removed, and the left gastric artery was removed. Then, the suprapyloric lymph nodes (No. 5), lymph nodes in front of the common hepatic artery (No. 8a), behind the common hepatic artery (No. 8p), in the hepatoduodenal ligament (along the hepatic artery) (No. 12a), along the splenic artery (No. 11), left gastric artery trunk (No. 7), and around the celiac artery (No. 9) were dissected.

Finally, the retrogastric space was fully expanded and dissociated upward to the end of the diaphragm on both sides.

The left gastroepiploic artery and vein were exposed at the tail of the pancreas. After ligation and transection, the gastric curvature lymph nodes (No. 4) and splenic hilar lymph nodes (No. 10) were dissected. The short gastric vessels were removed to the cephalic side, and the left cardiac lymph nodes (No. 2) were dissected. In addition, the right cardiac lymph nodes (No. 1) and the lesser curved gastric lymph nodes (No. 3) along the lesser curvature of the stomach were dissected.

The esophagus was dissociated from right to left, the phrenic esophageal ligament was cut open (Fig. 1c), the inferior cardiac bursa was exposed on the right, and the left diaphragm was removed from the upper and left sides of the esophagus, approximately 8 cm. Subsequently, the subphrenic lymph nodes (No. 19) and diaphragmatic hiatal lymph nodes (No. 20) were dissected.

#### Inferior mediastinal lymph node dissection

The upper boundary was the level of the inferior pulmonary vein (Fig. 1d), the lower boundary was the end of the diaphragm, the anterior boundary was the posterior wall of the pericardium, the posterior boundary was in front of the thoracic aorta, and the bilateral boundary was the inferior pulmonary ligament. The lower thoracic paraesophageal lymph nodes (No. 110) (Fig. 1e), supraphrenic lymph nodes (No. 111), and posterior mediastinal lymph nodes (No. 112) (Fig. 1f) were removed. The mesoesophagus was stripped nude at the level of the inferior pulmonary vein. Anatomical landmarks of the No. 110 lymph node were observed with respect to the caudal edge of the inferior pulmonary vein to the gastroesophageal joint, anatomical landmarks of the No. 111 lymph node were located above the top of the diaphragm, near, or behind the angle of the diaphragm, and anatomical landmarks of the No. 112 lymph node were detected in the lower lung ligament.

An intraluminal linear cutting occluder was placed from No. 5 trocar hole (Fig. 1g), and 3 cm of the esophagus was removed at the upper edge of the tumor. Then, OrVil esophagojejunostomy was performed.

#### Anastomosis method (Fig. 1h)

Total laparoscopic OrVil anastomosis was performed. The jejunum was cut off by 30 cm at the distal end of the flexion ligament, the stapler was inserted from the distal end, and the jejunum was penetrated through the mesenteric margin. The OrVil pedestal was placed through the incision, followed by endoscopic end-to-side esophagojejunostomy. The broken end of the jejunum was closed with a linear cutting occluder. All the anastomoses were sutured and strengthened by the laparoscopic seromuscular layer (Fig. 1). The jejunum, at 45–60 cm distal to the esophagojejunostomy, was anastomosed with the input loop jejunum.

#### Placement of the drainage tube

A closed thoracic drainage tube was placed through hole 5, and the left diaphragm was closed with an inverted stab suture. A double cannula was placed through hole No. 3 for drainage and inserted into the right mediastinum from the right side of the anastomosis. A double cannula was also placed through hole No. 1 for drainage and inserted into the left mediastinum along the left side of the anastomosis.

A jejunal nutrition tube was inserted 45 cm distal to the Brown anastomosis, led out through the left abdominal wall and fixed.

## Results

**Fig. 2:**
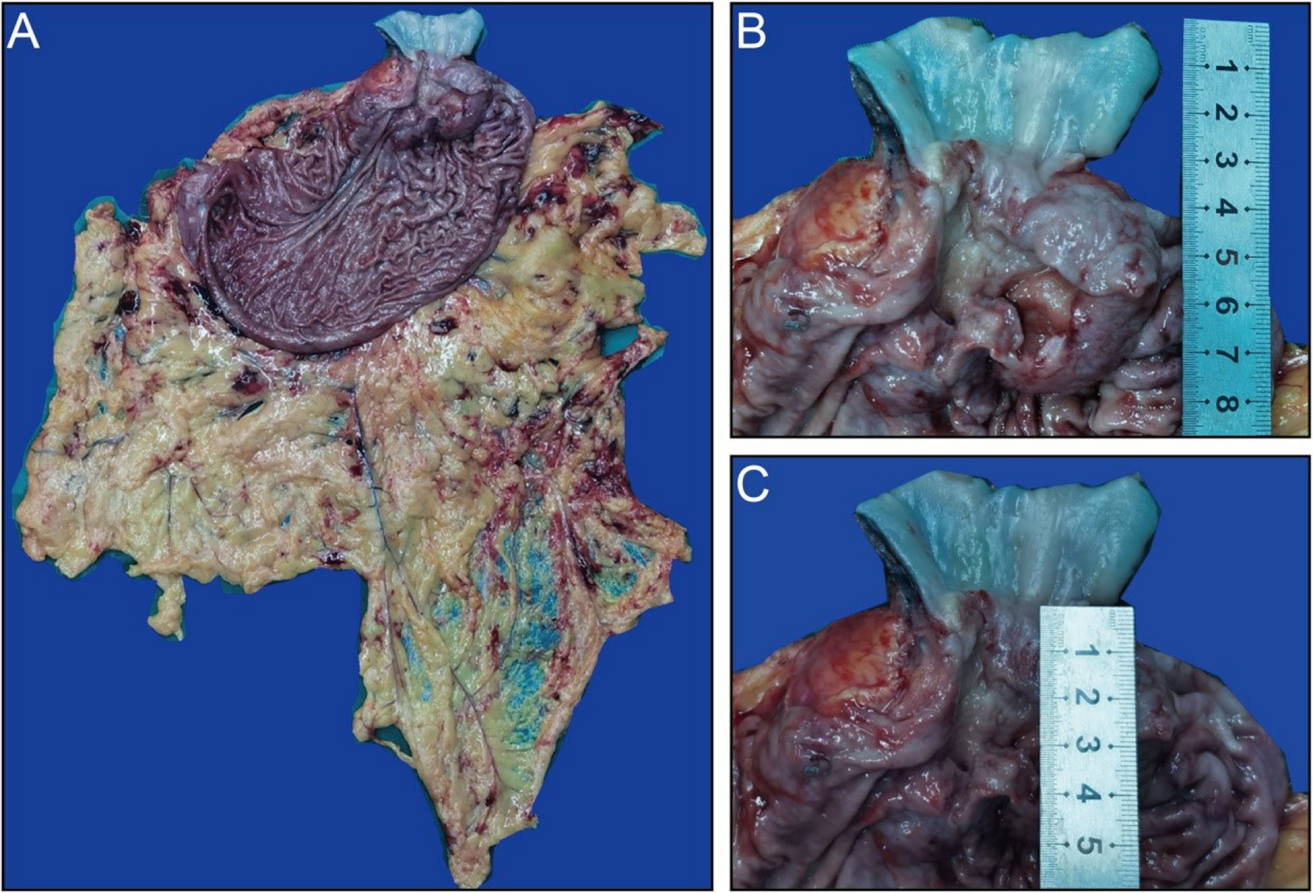
**a** Gross specimen. **b** Distance between the incisal margin of the esophagus and the tumor. **c** Distance between the lower margin of the tumor and the tooth line

From April 2019 to June 2019, 10 patients with Siewert II adenocarcinoma underwent total laparoscopic radical resection of Siewert II AEG through the left diaphragm and left thoracic auxiliary hole. Table 1 lists the clinical characteristics of the study participants. The cohort consisted of 7 males and 3 females, with a male-to-female ratio of 7:3. The average age of the participants was 60 years, the average body mass index (BMI) was 23.24, the average postoperative pathological tumor size was 7.5 cm (Fig. 2a), the largest tumor diameter was 11.5 cm, the average length of esophageal invasion under gastroscopy before surgery was 2.2 cm (Fig. 2b, c), and the preoperative pathological stage was mainly stage III. Table 2 shows the relevant characteristics of the researcher’s operation and pathology: the average duration of the operation was 348 ± 37.52 min, the longest operation time was 405 min, the shortest operation time was 340 min, the average dissection time of the lower mediastinum was 20.6 min, the average time of OrVil anastomosis in the thoracic cavity was 29.8 min, the average time of seromuscular suture through the left thoracic auxiliary hole was 11 min, and the average safety margin was 3.2 cm. The average total number of lymph nodes dissected was 40.6. Among these parameters, the average number of lower mediastinal lymph nodes dissected was 6.2. The lymph node metastasis rate of the N110 group was 9 ± 12.45%. Postoperative pathological stage III patients accounted for 90% of the cohort. Table 3 shows the intra- and postoperative short-term complications and postoperative recovery of the patients. The average intraoperative blood loss was 170 ± 57.47 mL, and no anastomotic leakage or anastomotic stenosis occurred. After the operation, 2 cases of pleural effusion and 1 case of pulmonary infection were detected by imaging findings; these were cured (as no fever) by conservative treatment, and no other related clinical symptoms were observed. The average time of intestinal function recovery was 2 days, and the first time of enteral nutrition through a jejunal nutrition tube was 2.4 days. Patients with stage III disease or above generally chose the Sox regimen as adjuvant chemotherapy for 6–8 cycles. No tumor recurrence was found in the 10 patients at 1 year after the operation, and follow-up work is still in progress.

**Table 1:** Characteristics of the study patients

	*N =* 10
Sex (male/female)	7/3
Age (years)	60 ± 7.5
BMI (kg/m^2^)	23.24 ± 2.62
Tumor size (cm)	7.5 ± 2.4
Length of esophageal invasion (cm) (under a gastroscope)	2.2 ± 0.45
cT stage (2/3/4)	2/4/4
cN stage (0/1/2/3)	2/4/3/1
c stage (IIB/III)	2/8

**Table 2:** Surgical and pathological results

	*N =* 10
Operation time (min)	348 ± 37.52
Dissection time of the lower mediastinum (min)	20.6 ± 6.84
Intrathoracic anastomosis time (min)	29.8 ± 13.05
Suture time of the anastomotic seromuscular layer (min)	11.2 ± 2.77
Safety margin	3.2 ± 0.84
Total number of lymph nodes dissected	40.6 ± 17.47
Number of lower mediastinal lymph nodes dissected	6.2 ± 4.09
Number of lymph nodes dissected at No. 110	3.4 ± 1.82
Lymph node-positive rate at No. 110 (%)	9 ± 12.45
pT stage (2/3/4a/4b)	2/3/5/0
pN stage (0/1/2/3)	1/2/5/2
p stage (II/IIIA/IIIB)	1/2/7

**Table 3:** Short-term complications during and after surgery and recovery

	*N =* 10
Intraoperative blood loss (mL)	170 ± 57.47
Anastomotic leakage (*n*)	0
Anastomotic stenosis (*n*)	0
Pleural effusion (*n*)	2
Pulmonary infection (*n*)	1
Time of intestinal function recovery (days)	2.2 ± 0.45
First time of enteral nutrition through a jejunal nutrition tube (days)	2.4 ± 0.55

## Discussion

### Feasibility of transabdominal mediastinal lymph node dissection of Siewert II AEG

At present, the classification of AEG is based on the Siewert classification proposed by the German scholar Siewert in 1987. Type II refers to the tumor center located from 1 cm above the dentate line to 2 cm below the dentate line [4]. As AEG metastasizes mainly through lymph nodes, a large number of studies [5–7] have confirmed that abdominal and mediastinal lymph node dissection is crucial for AEG. A study on the mediastinal lymph node metastasis rate of type II AEG showed that it was closely related to the length of the tumor invading the esophagus [8]. When the upper end of the tumor invaded 2 cm above the EGJ, the rate of lymph node metastasis in the lower mediastinum increased. When invasion was > 3 cm above the EGJ, the rate of lymph node metastasis in the upper-middle mediastinum increased. Therefore, for II AEG, when tumors invade < 2 cm above the EGJ, the lower mediastinal lymph nodes should be routinely dissected, but when tumors invade the esophagus > 3 cm, the middle and upper mediastinal lymph nodes should be dissected simultaneously. In the Japanese multicenter prospective study, mediastinal lymph node metastasis of Siewert II AEG was mainly below the mediastinum; N110 lymph node metastasis was a common occurrence, with a metastasis rate of 9.0%. The lymph node metastasis rates of N111 and N112 were 3.4% and 2.0%, respectively [9]. Therefore, it is speculated that lower mediastinal lymph node dissection can be performed only for AEG patients in whom the esophageal invasion distance is <3 cm. The addition of the auxiliary hole allows mediastinal dissection and enhances the visual field. Additionally, bilateral inferior pulmonary veins are fully exposed, thereby reducing the risk of intraoperative collateral injury. In this group, the number of lower mediastinal lymph nodes dissected was 6.2 ± 4.09, and the positive rate of lymph nodes in the N110 group was 9 ± 12.45%; these findings were consistent with those reported previously. The average time of dissection was 20.6 ± 6.84 min, which was much shorter than that of mediastinal lymph node dissection through the esophageal hiatus.

### Safety of anastomosis and pathology

The tumor location, narrow local space, and occlusion of the costal arch and diaphragm render lymph node dissection and digestive tract reconstruction extremely difficult during laparotomy for AEG. However, with the continuous development of laparoscopic equipment and technology, the magnifying effect of the technique and the advantage of a 30° visual angle in laparoscopy have greatly improved the visual field of traditional laparotomy. However, for patients with obesity, a high tumor location, and a large tumor, the laparoscopic transabdominal esophageal hiatus approach may not guarantee complete tumor resection or inferior mediastinal lymph node dissection. If it is converted to thoracotomy during the operation, the duration of the operation may be prolonged, and the operation risk may increase [10]. After incision of the left diaphragm and left pleura, the space of the lower mediastinum is further expanded, and the lymph nodes in the lower part of the esophagus and lower mediastinum are exposed. On the other hand, the change in posture introduced during thoracic Ivor–Lewis laparoscopy makes it difficult to judge the condition of the abdominal cavity, and mesangial torsion and mesangial hemorrhage are common events. Esophageal-jejunal anastomosis via the abdominal esophageal hiatus approach often fails to complete the anastomosis at a high site due to the narrow space. A retrospective study of Siewert II AEG with stage ≥cT2 showed that in 45 cases of positive incisal margins, the cutting edge distance in 91% of cases was <3 cm, which was a risk factor for a positive cutting edge [11]. Another retrospective study involving 505 cases of AEG demonstrated that a distance from the incisal margin of the esophagus in vitro >3.8 cm (approximately 5 cm in vivo) was an independent risk factor for prognosis [12]. The average distance between the upper incisal margin of the esophagus and the tumor (in vitro) achieved at our center was 3.2 ± 0.84 cm, and no mesangial blood circulation disturbance or mesangial torsion was detected in any of the patients. The intraoperative freezing and postoperative pathologies of the upper incisal margin were negative. The safe distance of resection has been well guaranteed.

Furthermore, the average time of OrVil anastomosis in this study was 29.8±13.05 min. Complete laparoscopic esophagojejunostomy has a clear field of vision for accurate anastomosis, which is convenient to observe mesenteric tension, blood circulation, and the direction of the small intestine. Reconstruction of the digestive tract during type II AEG requires a high amputation position. A circular stapler has a high anastomotic plane and hence is dominant. Strikingly, no intractable anastomotic stenosis was observed after OrVil anastomosis [13]. Our center employed intraoperative gastroscopy to confirm the absence of anastomotic leakage and stricture, and use of a circular stapler proved to be an effective method.

### Anastomotic reinforcement and suture and rapid postoperative recovery

Anastomotic fistula is the most serious postoperative complication of esophageal and cardiac cancer, with a high fatality rate of 44.7%. In a report of laparoscopic-assisted radical resection of AEG through the left chest, 6.8% of the patients had postoperative anastomotic leakage [14]. In another clinical study of 160 patients, the incidence of anastomotic leakage with reinforced sutures was lower than that with circular stapling alone [15]. A recent animal study also showed that the incidence of anastomotic leakage decreased after end-to-end anastomosis [16]. Therefore, if the seromuscular layer can be strengthened after thoracic anastomosis, the occurrence of postoperative anastomotic leakage can be reduced. In the early laparoscopic radical resection of gastroesophageal junction cancer by incision of the left diaphragm in our center, we found that if the position of esophagectomy was high, it was difficult to embed the seromuscular layer of the anastomosis after transabdominal esophagostomy and anastomosis. The average entrapment time of the seromuscular layer was 11.2 ± 2.77 min, which could be attributed to the long transabdominal operation path and excessive angle. After the addition of the left chest auxiliary hole between the 6th and 7th ribs of the left chest, esophageal amputation and anastomotic suture were performed simultaneously. The suture of the seromuscular layer of the anastomosis was firm, and the suture time of the seromuscular layer was significantly shorter than before. Since the seromuscular layer was sutured accurately, the incidence of anastomotic leakage decreased significantly, which encouraged the patients to eat early after the operation. The average eating time of this group was 2.2 ± 0.45 days, which was in line with the concept of rapid rehabilitation surgery.

### An auxiliary hole in the left chest cavity did not increase patient trauma

One of the common postoperative complications of Siewert II AEG is pleural effusion and pulmonary infection. Previously, a double cannula was placed in the left and right mediastina through the esophageal hiatus, which was drained from the abdomen. We found that the incidence of postoperative pleural effusion was 50%, and the incidence of pulmonary infection was 30%. Although the majority of patients can be cured by conservative treatment, 20% of patients need intubation under the guidance of B-ultrasound to drain the effusion. On the other hand, through the placement of a closed thoracic drainage tube through the left thoracic auxiliary hole and without any additional trauma, postoperative pleural effusion can be drained, and the incidence of pulmonary infection can be reduced significantly. In this cohort, 2 patients experienced pleural effusion. However, puncture to drain a small amount of effusion under the guidance of B-ultrasound was not required. One of the two patients had a lung infection and was cured by conservative treatment. The majority of the patients tolerated the drainage tube, while the remaining complained of obvious pain. The time of getting out of bed and the average hospital stay in this group were similar to those who underwent laparoscopic surgery for AEG through the diaphragmatic hiatus approach.

In summary, we found that total laparoscopic radical resection of Siewert II AEG through the left diaphragm and left chest auxiliary hole provides an improved surgical field of vision and large surgical space. It is convenient to complete lower mediastinal lymph node dissection, esophagojejunostomy, and anastomotic reinforcement suture under direct vision. Moreover, patients can obtain satisfactory oncology results. Many defects, such as changing posture during the operation, a narrow mediastinal space, and difficulty judging the abdominal cavity during anastomosis, can be avoided. In addition, patients can eat and get out of bed early after the operation, which reduces the occurrence of postoperative pulmonary infection and other complications, thereby deeming it to have good application prospects. Nonetheless, additional cases and prolonged follow-ups are required to obtain long-term survival data.

## Conclusion

Total laparoscopic radical resection through the left diaphragm and left thoracic auxiliary hole for Siewert II AEG patients is feasible and safe. Thus, it may be another method for patients with esophageal invasion <3 cm. We need more research and a larger number of patients to support this statement.

## Data Availability

Not applicable for this manuscript.
